# Supplementary material for: Protein Intake and Protein Quality Patterns in New Zealand Vegan Diets: An Observational Analysis Using Dynamic Time Warping
Source: Nutrients. 2025 May 26;17(11):1806. doi: 10.3390/nu17111806 (PMC12157289; doi:10.3390/nu17111806)
Supplement: Supplementary file 1 [file nutrients-17-01806-s001.zip › nutrients-3621218-supplementary/Supplementary data and codes.pdf]

Dtw code:

```
Protein.time.Bioadjusted$DayxPart <- interaction(as.factor(Protein.time.Bioadjusted$DocName), as.factor(Protein.time.Bioadjusted$Day))
day.part <- unique(Protein.time.Bioadjusted$DayxPart)

dtw_distances.protein.bio <- matrix(NA, nrow = length(day.part), ncol = length(day.part)) #for storage.
# a lower value represents greater similarity between time series

# pairwise comparison of participants' time series data using DTW


for (i in 1:length(day.part)) { #loops over each participant
  for (j in 1:length(day.part)) { #loops over each participant again - creating nested loops
    if(i!=j) { #ensure that DTW is not calculated for same participant against themselves
      ts1 <- Protein.time.Bioadjusted[(Protein.time.Bioadjusted$DayxPart == day.part[i]), "final_Diary_Protein.B"]
      ts2 <- Protein.time.Bioadjusted[(Protein.time.Bioadjusted$DayxPart == day.part[j]), "final_Diary_Protein.B"]
      dtw_distances.protein.bio[i,j] <- dtw(ts1, ts2)$normalizedDistance
    }
  }
  print(i)
}



dtw_distances.protein.bio[is.na(dtw_distances.protein.bio)] <- 0
dtw_df.protein.bio <- as.data.frame(as.table(dtw_distances.protein.bio))
colnames(dtw_df.protein.bio) <- c("Participant_1 x Day", "Participant_2 X Day", "dtw_distance")

silhouette_analysis <- fviz_nbclust(dtw_distances.protein.bio, hcut , method = "silhouette")
print(silhouette_analysis)

#for clustering and obtaining dendrogram
set.seed(123)
hc_results.protein.bio <- hclust(as.dist(dtw_distances.protein.bio), method = "ward.D2") #hclust plots as dendrogram
plot(hc_results.protein.bio)
plot(hc_results.protein.bio, hang = -1)
dendo <- as.dendrogram(hc_results.protein.bio)
coloured_dend <- color_branches(dendo, k = 3)
plot(coloured_dend)

#for cluster plot in a 2-d scale
dist_matrix <- as.dist(dtw_distances.protein.bio) #convert normalised distance matrix to a dist object

# Compute the MDS coordinates using the dtw distance matrix
mds_coordinates.prot <- cmdscale(as.dist(dtw_distances.protein.bio), k = 2)  # k = 2 for 2D scaling #raw coordinates at this point
mds_coordinates.prot <- as.data.frame(mds_coordinates.prot)  # Convert to dataframe
```

```
colnames(mds_coordinates.prot) <- c("Dim1", "Dim2")  # Rename columns


hc.cut.protein.bio <- hcut(dtw_distances.protein.bio, k = 3, hc_method = "ward.D2")
print(hc.cut.protein.bio)
hc.clust.protein.bio <- fviz_cluster(hc.cut.protein.bio, data = mds_coordinates.prot, ellipse.type = "convex") #fviz_cluster used scaled coordinates
print(hc.clust.protein.bio) #2-d map with distances
```
